# Original Approach for Automated Quantification of Antinuclear Autoantibodies by Indirect Immunofluorescence

**DOI:** 10.1155/2013/182172

**Published:** 2013-12-18

**Authors:** Daniel Bertin, Noémie Jourde-Chiche, Pierre Bongrand, Nathalie Bardin

**Affiliations:** ^1^Aix-Marseille Université, Laboratoire d'Immunologie, Pôle de Biologie, Hôpital de la Conception, Assistance Publique-Hôpitaux de Marseille, 147 boulevard Baille, 13005 Marseille, France; ^2^Aix-Marseille Université, Centre de Néphrologie et Transplantation Rénale, Hôpital de la Conception, Assistance Publique-Hôpitaux de Marseille, 13005 Marseille, France; ^3^Aix-Marseille Université, UMR INSERM 1067 & UMR CNRS 7333, Laboratoire “Adhésion et Inflammation,” 13288 Marseille Cedex 09, France; ^4^Aix-Marseille Université, INSERM UMR 1076, Laboratoire “Endothélium, Pathologies Vasculaires et Cibles Thérapeutiques,” Faculté de Pharmacie, 13385 Marseille Cedex 05, Marseille, France

## Abstract

*Introduction*. Indirect immunofluorescence (IIF) is the gold standard method for the detection of antinuclear antibodies (ANA) which are essential markers for the diagnosis of systemic autoimmune rheumatic diseases. For the discrimination of positive and negative samples, we propose here an original approach named Immunofluorescence for Computed Antinuclear antibody Rational Evaluation (ICARE) based on the calculation of a fluorescence index (FI). *Methods*. We made comparison between FI and visual evaluations on 237 consecutive samples and on a cohort of 25 patients with SLE. *Results*. We obtained very good technical performance of FI (95% sensitivity, 98% specificity, and a kappa of 0.92), even in a subgroup of weakly positive samples. A significant correlation between quantification of FI and IIF ANA titers was found (Spearman's *ρ* = 0.80, *P* < 0.0001). Clinical performance of ICARE was validated on a cohort of patients with SLE corroborating the fact that FI could represent an attractive alternative for the evaluation of antibody titer. *Conclusion*. Our results represent a major step for automated quantification of IIF ANA, opening attractive perspectives such as rapid sample screening and laboratory standardization.

## 1. Introduction

Antinuclear antibodies (ANA) are essential biological markers for the diagnosis [1], classification, and disease activity monitoring [2] of systemic autoimmune rheumatic diseases. Given this central role, ANA screening should be accurate and reproducible. For several decades, indirect immunofluorescence (IIF) on HEp-2 cells has been the reference technique for ANA testing. Although new available techniques [3, 4] such as ELISA or multiplexing solid phase technologies have been proposed to replace IIF, the American College of Rheumatology (ACR) still recommends IIF as the gold standard method for ANA detection [5]. The main drawback of this technique is IIF reading subjectivity, intra- and interlaboratory variabilities complicating the standardization expected in modern laboratories.

Recently, commercial automated systems for ANA IIF reading and interpretation have become available and were described in the literature [6–11]. Most of them are based on data mining and supervised machine learning methods [12]. In addition to their complexity, they share a common weakness in the detection of weak positivity.

In this work, we describe an original algorithm named Immunofluorescence for Computed Antinuclear antibody Rational Evaluation (ICARE) for automation of IIF ANA evaluation offering excellent analytical performance and an attractive quantitative approach for positive/negative discrimination. We assess the quantification of the fluorescence intensity as an alternative to antibody titer evaluation and validate our approach in a population of patients with systemic lupus erythematosus (SLE).

## 2. Materials and Methods

### 2.1. Patients and Serum Samples

We collected serum samples from 2 cohorts of patients: a “routine cohort” and a “SLE cohort”. The routine cohort comprised 237 consecutive serum samples sent to the Immunology Laboratory for ANA analysis with IIF for outpatients or patients hospitalized in the Departments of Internal Medicine, Rheumatology, Dermatology and Cardiology of the University Hospitals of Assistance Publique-Hôpitaux de Marseille (AP-HM). The SLE cohort comprised 25 consecutive SLE patients meeting the American College of Rheumatology (ACR) classification criteria and followed up in the Nephrology Department of AP-HM. For all the SLE cohort patients, anti-double stranded DNA (ds-DNA) antibody levels were measured in sera with fluorescence-enzyme immunoassay (EliA dsDNA; Phadia, Uppsala, Sweden; now part of Thermo Fisher Scientific). All sera were retrospectively obtained from a declared serum bank (Biobank DC 2012-1704). This study did not need ethical approval or consent.

### 2.2. Patients Characteristics

The routine cohort comprised 237 patients, 93 males and 144 females, with a mean age of 49.4 years (range 3–90 years) (Table 1). Based on visual IIF ANA analysis, this cohort was splitted into ANA negative (*n* = 103) and ANA positive (*n* = 134) sera. As expected, there were significantly more women in the ANA IIF positive group than in the ANA IIF negative group (70% versus 51%, *P* = 0.01).

In the positive ANA IIF group, several single and mixed fluorescence patterns were represented (91 speckled, 10 centromeric, 7 nucleolar, 5 homogenous, 5 nuclear dots, 3 mitotic spindle apparatus, 11 homogenous-speckled, and 2 homogenous-nucleolar). The fluorescence titers ranged from 100 to more than 800. To analyze more accurately the sensitivity for weak positive detection, the ANA IIF positive group was subdivided into weak positive ANA IIF (titer = 100, *n* = 49, 24 males and 25 females, mean age 56.4 years, range: 11–50) and positive ANA IIF (ANA titer ≥ 200, *n* = 85, 19 males and 66 females, mean age 49.9 years, range: 8–89)

The SLE cohort comprised 25 patients, 7 males and 18 females, with a mean age of 37.2 years (range 17–74 years) (Table 1). Except for one patient, all patients were ANA positive (11 homogenous-speckled, 13 speckled) with ANA IIF titers ranging from 200 to above 800. Eight patients were negative for anti-dsDNA antibodies (antibody level < 16 IU/mL) and seventeen patients were positive (range: from 16 IU/mL to above 379 IU/mL).

### 2.3. ANA Testing

ANA in patients' sera were detected by commercial ANA HEp-2 indirect immunofluorescence assay. Automated instrument (PhD system, Bio-Rad Laboratories, Hercules, CA) was used for IIF slide preparation. Samples diluted in phosphate buffered saline were incubated on HEp-2 cells fixed on glass slides (Kallestad HEp-2 Cell Line Substrate, 12 wells slides, Bio-Rad Laboratories, Hercules, CA) for 30 minutes at room temperature (RT). The screening dilution was 1 : 100. After washing, bound antibodies were detected by incubation with fluorescein isothiocyanate (FITC) conjugated sheep anti-human immunoglobulin (Kallestad FITC conjugate, Bio-Rad Laboratories, Hercules, CA) for 30 minutes at RT. Subsequently, slides were washed, embedded with a 4,6-diamidino-2-phenylindole (DAPI) containing medium (Vectashield, Vector Laboratories Inc., Burlingame, CA), and visually assessed with a fluorescence microscope (Leica DM1000, Leica Microsystems, Germany) by two experienced observers. For each sample, fluorescence pattern and titer were visually assigned in case of positivity. The visual cut-off titer was 100 corresponding to sera with weak positivity. Based on visual ANA analysis, three patient groups were created: positive ANA (titer ≥ 200), weakly positive ANA (titer = 100), and negative ANA groups. The titer was defined as the reciprocal of the highest dilution of serum that still shows immunofluorescent nuclear staining.

### 2.4. Image Capture

**Figure 1:**
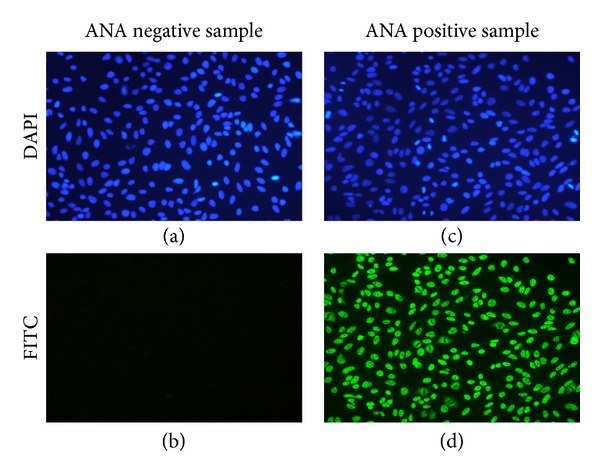
Examples of captured images. Examples of images obtained by IIF on HEp-2 cells from 2 different serum samples: one ANA negative ((a), (b)) and one ANA positive ((c), (d)) for both DAPI ((a), (c)) and FITC ((b), (d)) stainings. Objective: ×20.

For each patient, two images of the same central microscopic field were captured with 20x objective at two different excitation wavelengths: 480 nm for FITC stain and 360 nm for DAPI stain. Captures were performed with a fluorescence microscope (Leica DM1000, Leica Microsystems, Germany) equipped with 360 nm and 480 nm leds for excitation (FluoLed, Fraen corporation Srl, Settimo Milanese, Italy). Captures with 1392 × 1040 pixels resolution were performed with a color CCD camera (Infinity 2, Lumenera Corporation, Ottawa, Canada). Exposure times for FITC and DAPI captures were 200 ms and 300 ms, respectively. All captured color images were 24 bit-depth and have been saved in Tagged Image File Format (TIFF) for subsequent analysis. As an example, Figure 1 shows the IIF microscopic images obtained from one positive and one negative sera.

### 2.5. ICARE Algorithm Description for Image Analysis

First, using image analysis software, we splitted RGB color channels and kept blue or green channel for DAPI and FITC images, respectively. Then, DAPI image was used to determine nucleus position. This was performed using a thresholding method based on image histogram analysis. We defined the background intensity of DAPI image as the first peak of DAPI histogram. A threshold defined as twice this background intensity allowed appropriate segmentation and selection of nucleus region of DAPI image. This nucleus region selection was then superimposed on FITC image which allowed mean fluorescence intensity measurement of nucleus region of FITC image (MFI n). Then, an inversion of selection allowed mean fluorescence intensity measurement of non-nucleus background region of FITC image (MFI b).

### 2.6. ICARE Index Calculation

For each captured well, we defined a nondimensional index called fluorescence index (FI) and calculated as follows: FI = (MFI n)/(MFI b).

The reproducibility of FI was tested. A single sample with weakly positive ANA (titer = 100) was tested 10 times in 10 wells each day on 3 consecutive days. Coefficients of variation of FI on the 3 days were 8.6%, 8.7%, and 5.9%.

We also evaluated the effect of exposure time of the camera on FI by studying the FI as a function of the time-exposure (50–300 ms) for positive ANA patients (data not shown). No variation was observed attesting that FI values were time-exposure independent.

### 2.7. Statistics

Analytical performance of ICARE algorithm was evaluated by calculating sensitivity (Se.), specificity (Spe.), positive predictive value (PPV), and negative predictive value (NPV). Accuracy was defined as the proportion of the total number of correct predictions by FI. Mann-Whitney *U* test was used to compare the mean values of FI and Spearman's rank correlation coefficient to study the correlation between FI and IIF ANA titers. The agreement between visual and algorithmic interpretation was evaluated using Cohen's Kappa coefficient which takes on the value (i) zero if there was no more agreement between two tests than expected by chance (ii) 1 if there was a perfect agreement. Kappa values below 0.4 were considered as poor agreement, values between 0.4 and 0.75 as fair to good agreement, and values higher than 0.75 as excellent agreement as described [13]. Data were analyzed and curves plotted using R statistical software (R Foundation for Statistical Computing, Vienna, Austria) and Microsoft Excel 2007. The threshold for statistical significance was set at *P* = 0.05.

## 3. Results

### 3.1. Index Cut-Off Determination and Analytical Performance of ICARE Algorithm Performed on Routine Cohort

**Figure 2:**
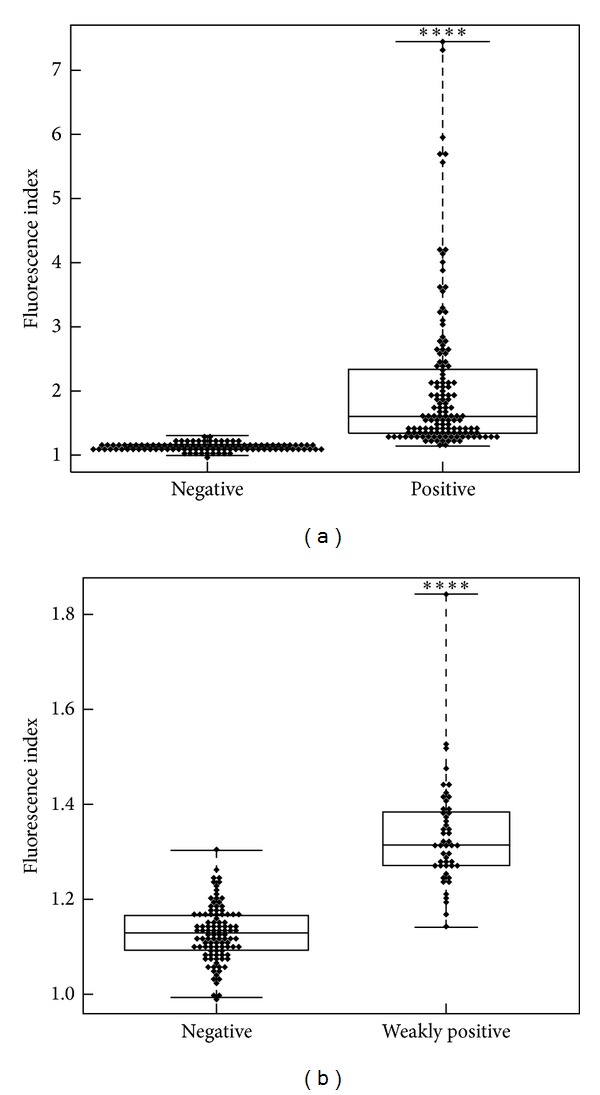
Fluorescence index in samples from the routine cohort (*n* = 237). (a) Comparison of samples with negative versus positive ANA in IIF. (b) Comparison of samples with negative versus weakly positive (titer = 100) ANA in IIF. Indexes were calculated with ICARE algorithm from captured IIF images and plotted for positive ANA patients (titer ≥ 100, *n* = 134), weakly positive ANA patients (titer = 100, *n* = 49), and negative ANA patients (titer < 100, *n* = 103). The box plots show the median value and range from the first to the third quartile. The whiskers extend between the maximum and the minimum indices. Indexes from ANA positive patients as well as those from weakly positive patients were significantly higher than those from ANA negative patients (*P* < 0.0001, Mann-Whitney *U* test).

FI was calculated for the 237 patients from the routine cohort. As shown in Figure 2(a), FI was significantly higher in ANA positive patients compared to ANA negative patients (mean value: 2.06 ± 1.18 versus 1.13 ± 0.06, *P* < 0.0001). To test further the ICARE algorithm performance in weakly positive samples (ANA titer of 100 corresponding to the visual cut-off), we compared samples with very low positive ANA to samples with negative ANA. Interestingly, FI was significantly higher in patients with weakly positive ANA than in patients with negative ANA (mean value: 1.33 ± 0.11 versus 1.13 ± 0.06, *P* < 0.0001) (Figure 2(b)).

**Figure 3:**
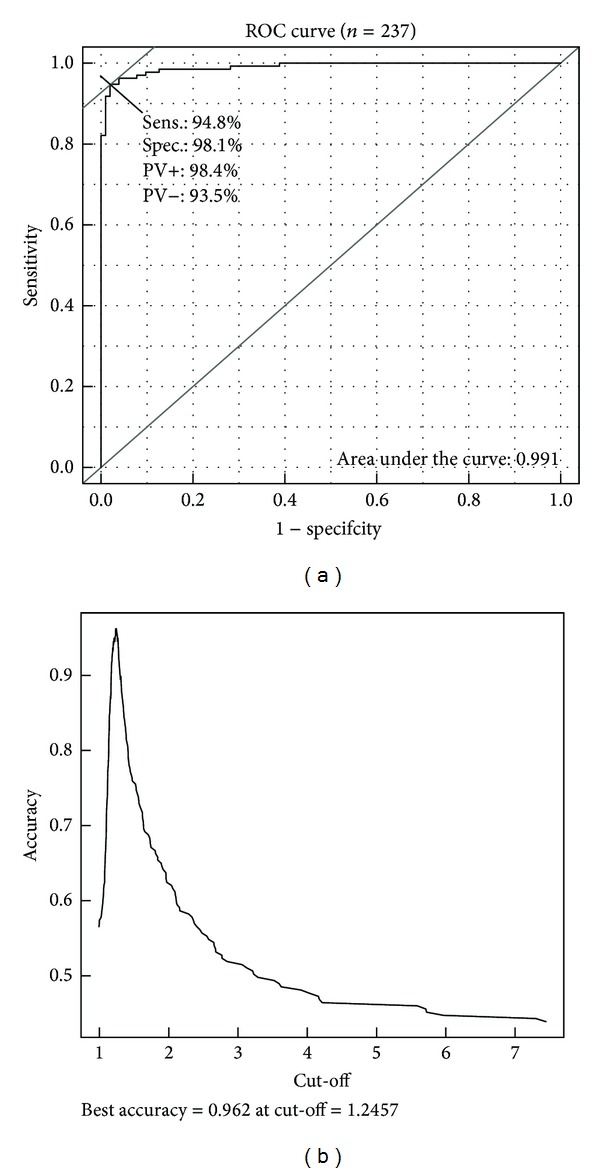
Cut-off determination for fluorescence index. (a) Receiver operating characteristic (ROC) of ANA screening with algorithm evaluation. Sens.: sensitivity, Spec.: specificity, PV+: positive predictive value, and PV−: negative predictive value. (b) Accuracy as a function of fluorescence index cut-off. Accuracy is the ratio between the number of correct prediction with algorithm and the total number of patients. Here the calculated cut-off is 1.246.

Cut-off determination of FI was performed using ROC analysis (Figure 3(a)) and accuracy curve (Figure 3(b)). FI cut-off value was set at 1.246 and area under the curve (AUC) was 0.991, attesting the excellent performance of the algorithm.

For the whole ANA IIF positive group (including weakly positive samples), sensitivity and specificity for positive/negative discrimination were, respectively, 95% and 98%. The concordance between visual and algorithmic evaluation was also excellent, with a Cohen's kappa of 0.923 (Table 2).

For weakly positive samples only, ICARE algorithm performance was also very good with 86% sensitivity and 98% specificity and a coefficient of concordance (Kappa) of 0.86.

### 3.2. Result Comparison between ICARE Algorithm and Visual ANA IIF

Concordant results between ICARE algorithm and visual evaluation of ANA by IIF were obtained for 228/237 routine samples (96%) (Table 3). The 9 remaining samples showed discrepant results: 2 were classified as weakly positive by the ICARE algorithm and negative by visual examination (false positive) and 7 were classified as negative by the ICARE algorithm but visually recognized as weakly positive by the expert (false negative). None of the false negative samples were associated with positive extractable nuclear antigen (ENA) or anti-dsDNA antibodies, and among them, 3 were drawn in an infectious context, 1 was from a 75-year-old patient and 1 from a patient treated for psoriatic arthritis with previously negative ANA. Importantly, no false negative was observed for samples with an ANA IIF titer ≥ 200.

### 3.3. Quantification of Fluorescence Index as an Alternative to ANA Titer

**Figure 4:**
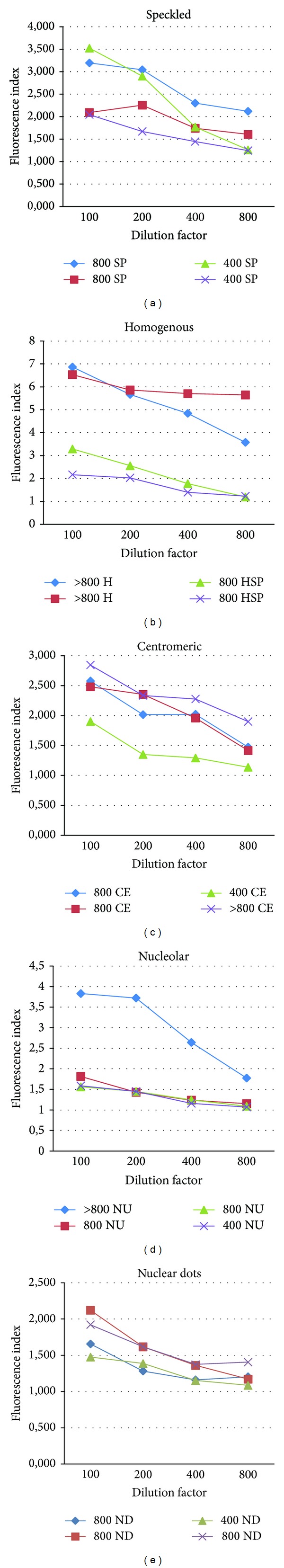
Study of fluorescence index as a function of dilution. Twenty ANA positive samples with different fluorescence patterns were diluted from 1 : 100 to 1 : 800. For each fluorescence pattern, four representative samples were diluted. For each sample, one curve represents the fluorescence index plotted as a function of dilution factor. SP: speckled, NU: nucleolar, H: homogenous, ND: nuclear dots, HSP: homogenous-speckled, and CE: centromeric.

**Figure 5:**
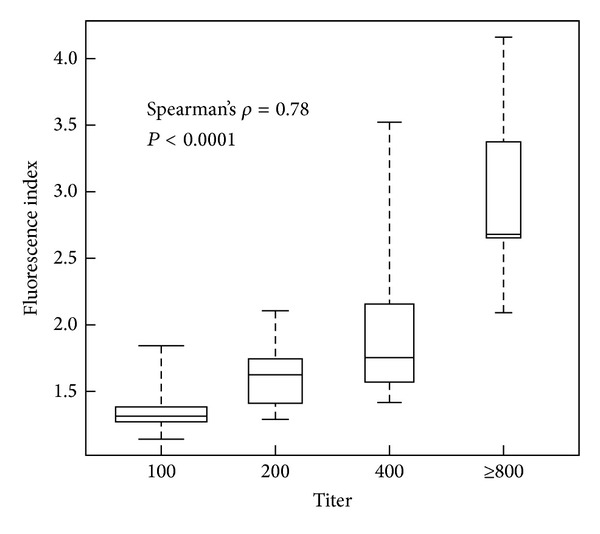
Correlation between fluorescence index and ANA titer. Calculated indices from patients with ANA positive speckled pattern (*n* = 87) were plotted as a function of the endpoint titer. The box plots show the median value and range from the first to the third quartile. The whiskers extend between the maximum and the minimum indices. There is a correlation between the fluorescence index and the fluorescence endpoint titer (Spearman's *ρ* = 0.78, *P* < 0.0001).

To assess the usefulness of FI quantitatively, as an alternative to antibody titer, we first investigated the effect of sample dilution on FI values. Twenty ANA positive samples with 5 different staining patterns (speckled, homogenous, centromeric, nucleolar, and nuclear dots) were diluted from 1 : 100 to 1 : 800. FI was evaluated for each dilution of a given sample (Figure 4). For all samples tested, a decrease in FI was obtained when the dilution factor increased, whatever the staining pattern tested. To comfort the relationship between FI values and antibody levels, we then analyzed FI value as a function of titer in 87 ANA positive speckled samples (Figure 5). A significant correlation between FI and ANA titers was found (Spearman's *ρ* = 0.78; *P* < 0.0001).

### 3.4. Clinical Validation on Patients with SLE

In order to evaluate the clinical performance of ICARE algorithm in connective tissue diseases, patients from the SLE cohort were tested. In agreement with our previous results, a significant correlation was found between FI and ANA titer (Spearman's *ρ* = 0.8; *P* < 0.0001) (Table 4). Interestingly, a significant correlation was also observed between FI and anti-dsDNA antibody levels in this cohort (Spearman's *ρ* = 0.47; *P* < 0.01).

## 4. Discussion

In this study we propose an original approach for automation of ANA IIF based on the calculation of a fluorescence index to discriminate positive and negative samples in a reproducible and nonobserver-biased way. We demonstrate excellent analytical performance of ICARE algorithm in comparison to the gold standard IIF visual method. Moreover, we show that FI could be used as a quantitative value to evaluate ANA titers. Last, we show that ICARE has potential interest in the monitoring of ANA in SLE patients.

Our approach is based on a quantitative strategy that mimics the routine analysis of ANA IIF. In the routine practice, the first step of this analysis consists in positive/negative screening that allows the rapid reporting of the 60–70% negative results requiring no further investigation. The second step is the pattern recognition, which is under development.

In the study, for the screening of 237 samples, ICARE reached a sensitivity of 95% a specificity of 98% and evidenced an excellent concordance with the visual method (accuracy: 96.2%, kappa = 0.923). To analyze more accurately the ICARE performance, analysis was performed on an ANA IIF subgroup presenting a weak positivity defining the visual cut-off (titer = 100). In this more difficult design, performance of ICARE was also very good with 86% of sensitivity, 98% of specificity, and an excellent coefficient of concordance (kappa = 0.86). The very good performance of ICARE suggests that it could replace the screening routine step by an automated approach.

Several commercially available systems are available for automated analysis of ANA by IIF: Aklides (Medipan, Berlin, Germany), G-Sight (Menarini, Florence, Italy), and EuroPattern (Euroimmun, Lübeck). In routine activity, good performance for ANA screening are reported. However, performance was not specifically evaluated in low positive ANA samples. This could change the interpretation of the results for the benefit of elevated positivity (high endpoint titers). Additionally, for Aklides system, Egerer et al. found a screening sensitivity of 94% for the whole population studied [6], while on the same system, Melegari et al. [7] found sensitivity of only 72% and suggested reassessing the cut-off for the detection of weakly positive samples. In the literature, the percentage of concordance between visual IIF and automated measures varied from 86% to 99%. With 96% of concordant results, our method is thus among the most performing. In our study, the only discrepant results (3.8%) were at the visual cut-off titer. The majority (7/9) were found weakly positive by the visual method. It is well known in laboratory practice that IIF visual reading becomes highly subjective and variable between observers when fluorescence intensity is around the cut-off titer. Moreover, none of the 7 samples were associated with positive extractable nuclear antigen (ENA) or anti-dsDNA antibodies, and other clinical settings than autoimmune disease may explain these visual low levels of ANA in some of them. Low titers of ANA may indeed be present in healthy aged subjects and patients with infections or with cancer [14]. This suggests higher performance of ICARE compared to visual methods and promotes an automated evaluation of ANA screening.

Only one system in the literature presents an “index,” but it is statistical, not quantitative, and is defined as a probability index. Indeed, for screening purpose, G-sight system provides a probability of positivity based on statistics of a set of previous training samples [10]. The fact that ICARE method, for automated evaluation of IIF ANA, was based on a quantitative evaluation opens attractive perspectives. We showed a significant correlation between the fluorescence index and ANA titers of the samples, suggesting that FI reflects the antibody level. Titer prediction with automated system could improve cost efficiency by suppressing the need of serial dilution and speeding up the report of the results. This quantitative index could give a comparative scale between laboratories allowing, in the future, a possible standardization of methods. Moreover, we validated this quantitative approach in a population of SLE patients. Interestingly, we also found a significant correlation between FI and anti-dsDNA antibody levels, which suggests a possible interest of FI in SLE disease activity monitoring.

In conclusion, the automated discrimination between positive and negative results represents a major step for automated evaluation of antinuclear autoantibodies by indirect immunofluorescence. Although ICARE algorithm should be tested in a multicenter analysis, it already presents several benefits such as the detection of weakly positive samples and a quantitative fluorescence index determination.

## Figures and Tables

**Table 1:** Characteristics of patients from the routine and SLE cohorts.

	ANA	*n*	Men	Women	Mean age (years)	Age std. (years)	Range (years)
Routine cohort (*n* = 237)	Negative	103	50	53	45.7	21.4	3–90
	Positive	134	43	91	52.3	18.7	8–89
	Titer = 100	49	24	25	56.4	18.7	11–50
	Titer ≥ 200	85	19	66	49.9	18.5	8–89

SLE cohort (*n* = 25)	Negative	1	0	1	n/a	n/a	n/a
	Positive	24	7	17	36.9	15.6	17–74

n/a: nonapplicable.

**Table 2:** Analytical performance of ICARE algorithm for ANA screening.

	Positive versus negative	Weakly positive versus negative
Sensitivity	94.5%	85.7%
Specificity	98.1%	98.1%
PPV	98.5%	95.5%
NPV	93.5%	93.5%
Kappa	0.923	0.861

PPV: positive predictive value, NPV: negative predictive value.

Kappa: Cohen's kappa coefficient.

**Table 3:** ANA screening agreement between visual and ICARE evaluations.

	ICARE+	ICARE−	Total
Visual+	127	7	134
Visual−	2	101	103

Total	129	108	237

**Table 4:** Correlations of FI with anti-dsDNA antibody levels and IFI titer.

Parameter	IFI titer	Anti-dsDNA
Spearman's *ρ*	0.80	0.47
*P* value (two-tailed)	<0.0001	0.02

## Abbreviations

ANA: Antinuclear antibodies
SLE: Systemic lupus erythematosus
IIF: Indirect immunofluorescence
FITC: Fluorescein isothiocyanate
DAPI: 4,6-Diamidino-2-phenylindole
FI: Fluorescence index
Se.: Sensitivity
Spe.: Specificity
PPV: Positive predictive value
NPV: Negative predictive value
ROC: Receiver operating characteristic
ENA: Extractable nuclear antigen
dsDNA: Double-stranded DNA.
